# Analysis of sickness absenteeism in a public university: a temporal statistical study

**DOI:** 10.47626/1679-4435-2026-1547

**Published:** 2026-04-28

**Authors:** Sara do Nascimento Cavalcante, Edmara Chaves Costa

**Affiliations:** 1 Universidade da Integração Internacional da Lusofonia Afro-Brasileira, Redenção, CE, Brazil

**Keywords:** absenteeism, risk factors, occupational health, university.

## Abstract

**Introduction:**

Due to the dynamic nature of work and the establishment of new universities in different locations with unique characteristics, researching the illnesses affecting their staff and their potential impact on working life is necessary.

**Objectives:**

To analyze the occurrence of sickness absenteeism among employees of a public university from 2013 to 2022.

**Methods:**

This ecological, descriptive, retrospective study adopted a quantitative approach and was conducted using data on sick leave among employees of a Brazilian university from 2013 to 2022. Based on these data, indicators were calculated to measure absenteeism. Descriptive and analytical statistics were applied, using the chi-square test. The study was approved by the institution’s research ethics committee.

**Results:**

During the study period, 1,010 sick leave cases were identified, totaling 26,406 days of absenteeism, indicating a growing trend, with peaks in 2019 and 2022 (p-value < 0.0168; correlation coefficient: 0.53). Fluctuations were observed in the difference-index-years and difference-frequency-years indicators between 2014-2015 and 2019-2020, along with a decrease in the duration index of sickness absenteeism. Approximately 68.02% of absences occurred among women, and 70.60% among employees performing administrative activities. Regarding the profile of illnesses, absences were most frequent in category 4 - diseases affecting the sensory organs (33.60%; n = 339; p < 0.000) and category 3 - psychological and neurological disorders (27.55%; n = 278; p < 0.000).

**Conclusions:**

Over the study period, levels of sickness absenteeism showed a tendency to increase, which highlights the need for greater institutional attention to employee health.

## INTRODUCTION

In the world of work, sickness absenteeism is a matter of concern, as it is associated with reduced institutional productivity and individual suffering [^1^]. Currently, it represents a growing issue, particularly among permanent employees and those in administrative roles [^2^], and constitutes a complex phenomenon often linked to poor health status, age, and workload [^3^,^4^] as well as to situations of organizational injustice [^1^] and workplace bullying [^5^].

Absenteeism affects not only the ill worker but also the entire network surrounding them, including the institution, management, coworkers, and family members, representing one of the main burdens for organizations [^6^]. It particularly impacts team performance and the quality of services provided [^7^], leading to organizational losses [^4^,^8^].

This study focuses on federal public service workers employed at universities. These higher education institutions generally include diverse professional categories, divided into two main groups: faculty and administrative staff. Careers in the federal public service grant employees job stability after 3 years of service, based on performance evaluation [^9^].

However, recent changes in legal provisions have altered some of the guarantees previously afforded to these workers, raising concerns about career prospects, particularly regarding retirement. These changes include the elimination of retirement based on length of service, with retirement now based solely on age, as well as the end of the right to retire with full salary for employees who entered public service after 2003 [^10^].

Studies have highlighted the influence of psychosocial factors at work as an important risk for the development of illness and, consequently, absenteeism [^11^]. In this context, analyzing absenteeism indicators has become increasingly relevant as an essential mechanism to understand workers’ health conditions and to support the development of strategies for health promotion and prevention in occupational health and safety. Such research provides a foundation for discussions on workers’ health conditions and for the development of policies aimed at health promotion, prevention, and rehabilitation [^2^,^12^,^13^].

Therefore, this study is justified by the characteristics of the institution under investigation, as it is a university that presents particular challenges in the work dynamics of its employees, given that it is relatively new compared to others in the regional context in which it operates. In addition, it has the distinctive feature of hosting international students and being located in the interior regions of its states. Accordingly, this study aims to analyze the occurrence of sickness absenteeism among employees of a public university from 2013 to 2022.

## METHODS

This is an ecological, descriptive, retrospective study with a quantitative approach, conducted using secondary data on health-related leave among all employees (faculty and administrative staff) of a Brazilian higher education institution located in the states of Bahia and Ceará, from 2013 to 2022.

Data provided by the institution were extracted from *Subsistema Integrado de Atenção à Saúde do Servidor* (SIASS) and did not contain information that could identify individual employees. Data were organized and tabulated using Microsoft Excel^®^, in order to structure the following variables: type of leave, disease classification code according to the International Classification of Diseases, 10^th^ Revision (ICD-10), number of days of leave, and department of assignment. The number of working days per year was calculated using an online tool that estimates annual working days and holidays [^14^].

The absenteeism indicators proposed by Ramalho [^15^] were used: sickness absenteeism index (total number of sick leave [SL] days/number of workers × working days), which indicates the percentage of lost workdays due to sick leave; frequency of SL (total number of SL/total number of workers), which represents the average number of sick leave episodes per worker; frequency of workers with SL (number of workers with SL/total number of workers), which represents the proportion of workers on sick leave; duration index of sickness absenteeism (total number of SL days/number of SL episodes), which indicates the average number of days per sick leave episode; and severity index (total number of SL days/total number of workers), which indicates the average number of days of absence per worker.

Data processing was performed using the public-domain software Epi Info™, version 7.2.5.0 (CDC, Atlanta, GA, USA), and IBM SPSS Statistics for Windows, version 23 (IBM Corp., Armonk, NY, USA).

Data were grouped into six categories based on similarities among ICD-10 chapters: Category 1 - diseases triggering immune responses (Chapters I [A00-B99] and II [C00-D48]); Category 2 - hematological, circulatory, and endocrine diseases (Chapters III [D50-D89], IV [E00-E90], and IX [I00-I99]); Category 3 - psychological and neurological disorders (Chapters V [F00-F99] and VI [G00-G99]); Category 4 - diseases affecting the sensory organs (Chapters VII [H00-H59], VIII [H60-H95], X [J00-J99], XI [K00-K93], XII [L00-L99], XIII [M00-M99], and XIV [N00-N99]); Category 5 - conditions related to maternal and child health and genetics (Chapters XV [O00-O99], XVI [P00-P96], and XVII [Q00-Q99]); Category 6 - conditions related to health status and external causes (Chapters XVIII [R00-R99], XIX [S00-T98], XX [V01-Y98], XXI [Z00-Z99], and XXII [U00-U85]).

During the results description, descriptive analysis of variables was performed, with data presented as absolute and relative frequencies. The prevalence of health-related leave among employees across the time series was analyzed based on independent variables, presenting outcome proportions and differences in percentage points between years. Pearson’s chi-square test was used to assess differences in proportions. A significance level of 5% was adopted for all analyses. Results were presented in tables and a graph.

Although this study used secondary data without the possibility of identifying individuals, prior approval by the institution’s research ethics committee was required, as the data were not publicly available. The study was approved under CAAE no. 39390220800005576 and opinion no. 4,429,653.

## RESULTS

A documentary analysis of health-related leave data among employees of the institution, extracted from SIASS, identified 1,010 sick leave episodes between 2013 and 2022, totaling 26,406 days of absenteeism.

For the calculation of the sickness absenteeism index, duration of sickness absenteeism, and severity index, according to Ramalho [^15^], data on the number of employees at the institution [^16^] were also aggregated, and the number of working days per year was calculated using an online tool (Table 1). The index results were expressed as percentages.

**Table 1:** Data on variables related to sickness absenteeism indices in a public higher education institution. Brazil, 2025

Year	No. of workers with sick leave	Total no. of sick leave episodes	Total no. of sick leave days	Total no. of workers in the institution	Working days per year
2013	19	24	565	237	255
2014	43	50	1.417	419	255
2015	28	28	1.005	523	252
2016	68	83	2.670	622	253
2017	112	128	3.054	665	251
2018	122	130	3.019	706	252
2019	158	179	4.000	712	255
2020	63	69	1.729	721	253
2021	93	108	3.979	733	253
2022	199	211	4.968	737	253

Source: Data collected from the study (2025).

**Figure 1:**
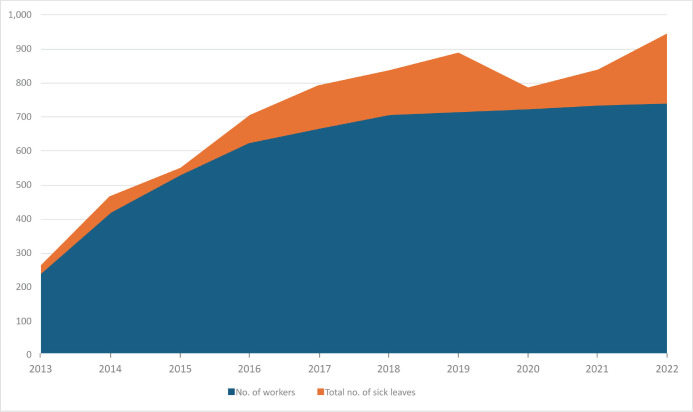
Representation of the number of workers and the number of sick leave episodes per year from 2013 to 2022 (p-value < 0.0168; correlation coefficient: 0.53).

Based on the application of the formulas, the results were presented in Table 2, showing that, over the decade, sick leave exhibited a growth pattern (p < 0.0168; correlation coefficient: 0.53), reaching peak values in 2019 and 2022 (Figure 1).

**Table 2:** Measures and indices for assessing absenteeism in the time series from 2013 to 2022 in a public higher education institution. Brazil, 2025

Year	SAI^1^	diy^2^	FSL^3^	dfy^4^	FWSL^5^	diy	SADI^6^	dfy	SI^7^	diy
2013	0,93	-	10,13	-	8,02	-	23,54	-	2,38	-
2014	1,33	0,39	11,93	1,81	10,26	2,25	28,34	4,80	3,38	1,00
2015	0,76	-0,56	5,35	-6,58	5,35	-4,91	35,89	7,55	1,92	-1,46
2016	1,70	0,93	13,34	7,99	10,93	5,38	32,17	-3,72	4,29	2,37
2017	1,83	0,13	19,25	5,90	16,84	5,91	23,86	-8,31	4,59	0,30
2018	1,70	-0,13	18,41	-0,83	17,28	0,44	23,22	-0,64	4,28	-0,32
2019	2,20	0,51	24,72	6,31	22,19	4,91	22,72	-0,51	5,62	1,34
2020	0,95	-1,25	9,71	-15,01	6,93	-15,26	24,70	1,98	2,40	-3,22
2021	2,18	1,23	14,87	5,16	10,91	3,98	37,06	12,36	5,51	3,11
2022	2,66	0,49	29,17	14,30	27,00	16,09	23,11	-13,95	6,74	1,23
t^8^		↑**1,73**		**↑19,05**		**↑18,98**		**↓**-0,43		**↑4,36**

1 SAI = sickness absenteeism index.

2 diy = difference in index between years.

3 FSL = frequency of sick leave.

4 dfy = difference in frequency between years.

5 FWSL = frequency of workers with sick leave.

6 SADI = sickness absenteeism duration index.

7 SI = severity index.

8 t = cumulative trend.

Source: Data collected from the study (2025).

However, fluctuations were observed in the difference-in-index-between-years and difference-in-frequency-between-years indicators between the periods 2014 to 2015 and 2019 to 2020. Regarding differences in the sickness absenteeism index, frequency of sick leave, frequency of workers on sick leave, and severity index, despite annual variations, an upward trend was observed in the cumulative analysis. In contrast, the duration index of sickness absenteeism showed a decrease (-0.43), indicating a slight reduction in the average number of days per leave episode (Table 2).

When analyzing the sex variable in aggregate, a higher prevalence of sick leave was observed among women (68.02%; n = 687). However, when each stratum was analyzed separately, the differences in percentage points between sexes were not substantial. Similarly, the analysis of the predominant activity variable showed a higher number of sick leave episodes in administrative roles (70.60%; n = 713), with a growth trend observed when considering the cumulative differences in percentage points between activity groups (Table 3).

**Table 3:** Distribution of sex and predominant activity variables in relation to absenteeism in the time series from 2013 to 2022 in a public higher education institution. Brazil, 2025

YEAR	SEX						PREDOMINANT ACTIVITY					
	Female			Male			Administrative			Teaching		
	f	%	p.p.^1^	f	%	p.p.	F	%	p.p.	f	%	p.p.
2013	17	2.47	-	7	2.17	-	17	2.38	-	7	2.36	-
2014	35	5.09	2.62	15	4.64	2.48	41	5.75	3.37	9	3.03	0.67
2015	21	3.06	-2.04	7	2.17	-2.48	20	2.81	-2.95	8	2.69	-0.34
2016	54	7.86	4.80	29	8.98	6.81	70	9.82	7.01	13	4.38	1.68
2017	81	11.79	3.93	47	14.55	5.57	106	14.87	5.05	22	7.41	3.03
2018	91	13.25	1.46	39	12.07	-2.48	106	14.87	0.00	24	8.08	0.67
2019	120	17.47	4.22	59	18.27	6.19	119	16.69	1.82	60	20.20	12.12
2020	43	6.26	-11.21	26	8.05	-10.22	52	7.29	-9.40	17	5.72	-14.48
2021	76	11.06	4.80	32	9.91	1.86	28	8.13	0.84	50	16.84	11.11
2022	149	21.69	10.63	62	19.20	9.29	124	17.39	9.26	87	29.29	12.46
p-value^2^	> 0,000						< 0,000					

Legend:

1 p.p. = Difference in percentage points.

2 Pearson’s chi-square test of independence.

Source: Data collected from the study (2025).

Regarding the types of diseases leading to sick leave, the 22 chapters of the ICD-10 were used, according to data from SIASS, with the addition of a category for cases without ICD classification.

Chapter V - mental and behavioral disorders (F00-F99) (25.64%; n = 259) accounted for the highest number of sick leave episodes throughout the time series, followed by Chapter XIII - diseases of the musculoskeletal system and connective tissue (M00-M99) (10.30%; n = 104) and Chapter X - diseases of the respiratory system (J00-J99) (9.31%; n = 94), both showing a growth trend. In contrast, Chapter XIX - injury, poisoning, and certain other consequences of external causes (S00-T98) (-20.73) and Chapter IX - diseases of the circulatory system (I00-I99) showed negative variation (-13.35).

As expected, the groups of mental and behavioral disorders (F00-F99), diseases of the musculoskeletal system and connective tissue (M00-M99), and diseases of the respiratory system (J00-J99) were the most prevalent, both in number of sick leave episodes and recurrence throughout the time series (2013-2022). In addition, Chapter I - certain infectious and parasitic diseases (A00-B99), Chapter XXI - factors influencing health status and contact with health services (Z00-Z99), Chapter XI - diseases of the digestive system (K00-K93), and Chapter XV - pregnancy, childbirth, and the puerperium (O00-O99) were present throughout the entire time series.

Given the number of ICD-10 chapters, for analytical purposes, they were grouped into subcategories based on similarity criteria, resulting in six categories. After data processing, the highest number of sick leave episodes was concentrated in Category 4 - diseases affecting the sensory organs (33.60%; n = 339; p < 0.000) and Category 3 - psychological and neurological conditions (27.55%; n = 278; p < 0.000).

With the organization of these categories, the time series showed occurrences of diseases in most years, except for Category 2 - hematological, circulatory, and endocrine diseases, which had no records in 2020. Considering the grouping of sick leave by year of occurrence, statistical significance was observed (p < 0.000). Over the decade, no category showed a decreasing trend; however, Categories 6 (27.53), 4 (17.99), and 3 (17.72) stood out due to the largest variations in percentage points during the period (Table 4).

**Table 4:** Classification of the time series by homogeneous groups according to the International Classification of Diseases codes among employees of a higher education institution. Brazil, 2025

YEAR	CATEGORIES					
	1	2	3	4	5	6
	Row%^1^ (p.p.)^2^	Row% (p.p.)	Row% (p.p.)	Row% (p.p.)	Row% (p.p.)	Row% (p.p.)
2013	4.17	16.67	8.33	20.83	12.50	37.50
2014	4.17 (2.13)	16.67 (-2.44)	8.33 (2.53)	20.83 (2.65)	12.50 (7.25)	37.50 (1.69)
2015	6 (-1.06)	6 (0.00)	20 (-1.90)	28 (-2.06)	16 (-5.80)	24 (-2.25)
2016	7.14 (3.19)	10.71 (-2.44)	14.29 (9.18)	25 (5.90)	14.29 (1.45)	28.57 (1.12)
2017	6.10 (19.15)	2.44 (4.88)	40.24 (-0.32)	32.93 (3.54)	6.10 (4.35)	12.20 (1.12)
2018	17.97 (-14.89)	3.13 (9.76)	25 (1.90)	30.47 (3.24)	6.25 (0.00)	17.19 (2.81)
2019	6.92 (5.32)	6.15 (-4.88)	29.23 (2.22)	38.46 (7.37)	6.15 (8.70)	13.08 (4.49)
2020	8.70 (-8.51)	0 (-14.63)	30.43 (-7.59)	43.48 (-13.27)	2.90 (-17.39)	14.49 (-8.43)
2021	14.81 (10.64)	3.70 (9.76)	34.26 (16.46)	24.07 (-1.18)	7.41 (8.70)	15.74 (3.93)
2022	7.11 (-1.06)	3.32 (7.32)	26.54 (-4.75)	31.28 (11.80)	4.27 (1.45)	27.49 (23.03)
Total (t)^3^	9.32 (14.89)	4.06 (7.32)	27.55 (17.72)	33.60 (17.99)	6.84 (8.70)	18.63 (27.53)
p-value^4^	< 0.000					

1 Row% = Percentage of the row proportion.

2 p.p.= Difference in percentage points.

3 t = Cumulative trend.

4 Pearson’s chi-square test of independence.

Source: Data collected from the study (2025).

## DISCUSSION

Since work is an integral part of individuals’ lives and can directly impact their physical and mental health, sickness absenteeism represents one of the types of work absence that most contributes to understanding organizational dynamics, as its analysis provides insights into the workers’ health-disease process [^17^].

In this study, sickness absenteeism data from 2013 to 2022 among employees of a Brazilian university were analyzed. The findings suggest a possible increase in this phenomenon within this population, even with new hires over the years. Nevertheless, the sickness absenteeism index, which represents the percentage of working days lost due to illness, remained at low levels during the period (below 2.5%) [^18^], contrasting with studies involving other Brazilian public servants that reported higher rates [^2^,^19^].

Regarding differences in the indicators (i.e., sickness absenteeism index, sick leave frequency [18.50], frequency of workers with sick leave [18.98], and severity index [4.36]) despite annual variations, a cumulative upward trend was observed. In contrast, the duration index, which represents the average number of days per sick leave episode, showed a negative variation over time, indicating a reduction in the average duration of sick leave.

The sociodemographic characteristics of public servants on sick leave are unique and reflect the epidemiological profile of the analyzed period. Therefore, the multiple factors influencing their living conditions, including social, economic, and environmental aspects, must be considered [^19^].

A higher occurrence of sick leave was observed among women, consistent with national and international estimates regarding the prevalence of absenteeism [^2^,^8^,^19^-^21^]. This finding may be associated with a combination of biological, psychosocial, and cultural factors, such as the balance between work and family responsibilities, as well as workplace inequalities that impose greater emotional demands and a greater need for support [^19^].

Work involves diverse and context-specific characteristics that evolve with changes in society, technology, and institutional priorities [^22^]. Sickness absenteeism has increased in recent years. In the present study, university employees performing administrative roles showed a higher number of sick leave episodes, which is consistent with findings from other studies involving public servants [^2^,^20^].

However, Atz & Remor [^20^] highlight the risk of underreporting sick leave among faculty members due to the lack of mandatory attendance control, omissions in record-keeping, and greater autonomy and flexibility in planning activities. Because of the nature of academic work, the need to meet performance targets may promote behaviors that normalize working while ill (presenteeism), driven by concerns about overburdening colleagues, compromising services, or the lack of replacement staff [^23^].

The analysis of absenteeism allows for understanding its impact not only on affected workers but also on their teams and the institution. This phenomenon is inversely associated with job satisfaction [^24^] and productivity [^4^], and directly related to financial burden [^8^], requiring management strategies involving both institutional leadership and health teams.

Thus, it is essential to develop mechanisms to assess the influence of workplace relationships on absenteeism and to encourage the implementation of safer work environments, free from overload and with adequate conditions for task performance [^3^].

Some studies emphasize the importance of analyzing job types and associated activities in relation to both the predisposition to illness and the occurrence of absenteeism [^25^]. Other authors highlight the interaction between economic, social, and health factors, which may trigger cascading effects, such as the need for multiple jobs, reduced rest time, increased stress, and impaired health [^4^].

As observed in this study, mental and behavioral disorders (F00-F99) represent the leading cause of sick leave among the analyzed employees. Similar to findings in other public service studies, mental health conditions pose a major challenge for organizations due to their impact on absenteeism. This scenario reinforces the need for continuous monitoring and strategies aimed at understanding work-related illnesses, in order to guide occupational health actions and support the development of institutional policies that promote health and quality of life [^19^-^21^,^26^,^27^].

Corroborating these findings, Atz & Remor [^20^] identified that occupational stress and negative health perception are associated with a greater number of sick leave days. Similarly, studies indicate a predominance of stress-related neurotic disorders and mood disorders, both associated with increased absenteeism [^21^]. Another relevant condition is burnout syndrome, which is related to high workloads and workplace conflicts and has been increasing, significantly impacting absenteeism [^26^].

Second, in this time series, diseases of the musculoskeletal system and connective tissue (M00-M99) (Chapter XIII) also stood out. In the study by Corrêa & Oliveira [^19^], these findings appear reversed, with these diseases representing the leading cause of sick leave, followed by mental disorders (Chapter V). In this context, Skovlund et al. [^28^] emphasize the importance of analyzing predictors of absence, particularly those related to musculoskeletal pain in regions such as the neck, shoulders, and lower back, which are more strongly associated with absenteeism.

Finally, diseases of the respiratory system (J00-J99) were among the most prevalent, both in the number of sick leave episodes and in their occurrence across all years of the studied time series (2013-2022), with a higher concentration during the COVID-19 pandemic period. Considering that the pandemic period brought multiple impacts on people’s lives, the world of work was significantly affected, both economically [^29^] and structurally, requiring immediate changes in work organization and promoting rapid shifts in workers’ daily routines, especially with the adoption of protective measures, social distancing, and, notably, home office [^30^].

The abrupt need to adapt to home office significantly affected job satisfaction among faculty members, being directly associated with changes in work routines and teaching practices, which led to several consequences for these professionals [^17^].

When analyzing the “pandemic effect,” it is observed that, in 2022, there was an increase in the frequency of several conditions, particularly respiratory and mental disorders, which may be related both to COVID-19 and to the indirect effects of the pandemic on professionals’ lives. This period also coincided with the gradual return to in-person activities at the institution.

The post-pandemic scenario intensified demands related to work organization and productivity, while also allowing greater flexibility through the adoption of hybrid work arrangements (in-person and home office), which requires attention regarding this new labor configuration [^29^].

Solutions highlighted in the literature include the development of public policies that ensure safe, dignified working conditions free from social inequalities, providing professionals with a healthy work environment [^3^,^27^]. These initiatives support professional development, well-being, and job satisfaction.

Accordingly, it is essential to develop workplace surveillance strategies focused on preventing health problems, improving comfort during work activities, and reducing exposure to occupational risks [^27^]. Likewise, promoting healthy habits (e.g., proper nutrition, hydration, and physical activity) is fundamental, considering the specific characteristics of each professional group [^13^,^27^].

Some limitations of this study should be considered. The number of sick leave episodes analyzed corresponds to formal records in SIASS, which may result in underreporting. Therefore, the inferences of this study are based on the available data, and estimates of work loss may not fully reflect the reality of the time series.

## CONCLUSIONS

As observed, throughout the time series of health-related leave at the institution from 2013 to 2022, despite fluctuations between 2014 and 2015 and between 2019 and 2020, sickness absenteeism levels showed an upward trend, reaching higher values in 2019 and 2022. The most recurrent disease groups were mental and behavioral disorders, diseases of the musculoskeletal system and connective tissue, and diseases of the respiratory system.

Thus, the importance of analyzing these indicators and implementing measures that promote health and quality of life is emphasized, with the aim of reducing sickness absenteeism rates within the institution. In addition, considering the post-pandemic scenario and the expansion of home office, relevant gaps are identified for future research on sickness absenteeism in this new context.

## Data Availability

The data cannot be made publicly available due to legal or ethical reasons.
